# The Mechanism for Primordial Germ-Cell Migration Is Conserved between Japanese Eel and Zebrafish

**DOI:** 10.1371/journal.pone.0024460

**Published:** 2011-09-12

**Authors:** Taiju Saito, Rie Goto-Kazeto, Yutaka Kawakami, Kazuharu Nomura, Hideki Tanaka, Shinji Adachi, Katsutoshi Arai, Etsuro Yamaha

**Affiliations:** 1 Nanae Fresh Water Laboratory, Field Science Center for Northern Biosphere, Hokkaido University, Nanae, Japan; 2 Laboratory of Aquaculture Genetics & Genomics, Faculty of Fisheries Sciences, Hokkaido University, Hakodate, Japan; 3 National Research Institute of Aquaculture, 22-1 Nakatsuhamaura, Minami-ise, Mie, Japan; 4 Laboratory of Aquaculture Biology, Faculty of Fisheries Sciences, Hokkaido University, Hakodate, Japan; University of Colorado, Boulder, United States of America

## Abstract

Primordial germ cells (PGCs) are segregated and specified from somatic cells during early development. These cells arise elsewhere and have to migrate across the embryo to reach developing gonadal precursors. Several molecules associated with PGC migration (i.e. *dead-end*, *nanos*1, and *cxcr*4) are highly conserved across phylum boundaries. However, since cell migration is a complicated process that is regulated spatially and temporally by multiple adaptors and signal effectors, the process is unlikely to be explained by these known genes only. Indeed, it has been shown that there are variations in PGC migration pattern during development among teleost species. However, it is still unclear whether the actual mechanism of PGC migration is conserved among species**.** In this study, we studied the migration of PGCs in Japanese eel (*Anguilla japonica*) embryos and tested the migration mechanism between Japanese eel and zebrafish (*Danio rerio*) for conservation, by transplanting eel PGCs into zebrafish embryos. The experiments showed that eel PGCs can migrate toward the gonadal region of zebrafish embryos along with endogenous PGCs, even though the migration patterns, behaviors, and settlements of PGCs are somewhat different between these species. Our results demonstrate that the migration mechanism of PGCs during embryonic development is highly conserved between these two distantly related species (belonging to different teleost orders).

## Introduction

Grafting and cell transplantation experiments between species have provided novel insights into developmental mechanisms of evolutionary change. In 1932, Speman and Schotte transplanted flank ectoderm derived from an early frog gastrula into the presumptive oral ectoderm regions of newt embryos [1]. They also transplanted flank ectodermal tissues from newt gastrula to the region of a frog gastrula destined to become the oral cavity. Reciprocal transplantation experiments revealed that the donor tissue can respond to the induction signals from the recipient and differentiate into the mouth structure with donor**-**specific morphology [1]. These results indicate that the mechanisms to develop mouth tissue in the embryo are conserved across species. Supporting this observation, it has been found that there are many genes that are conserved between protostomes and deuterostomes, with the homologs performing the same function. For example, the *Pax*6 gene from mouse and *Drosophila* induces ectopic eye development in fly and *Xenopus* embryos, respectively [2], [3].

Primordial germ cells (PGCs) are the precursors of germ cells in the embryo. In many animals PGCs do not arise within the gonad, but rather arise elsewhere and migrate across the embryo to reach developing gonadal precursors that form the ovaries in females and testes in males. In some animals (e.g., zebrafish, chicken, mouse, and *Xenopus*), it has been shown that the chemoattractant system consisting of *sdf-*1 (Chemokine ligand 12) and *cxcr*4 (alpha-chemokine receptor specific for SDF-1) play an important role in the migration of PGCs [4]–[10]. These observations suggest that at least some part of the migration mechanism of PGC is widely conserved among animal species. However, it is not clear how much of the mechanism of PGC migration is conserved across species.

In teleosts, the migration of PGCs has been well studied in zebrafish (*Danio rerio*). PGC migration in zebrafish takes place during the first 24 hours of its embryonic development [11], [12]. PGCs form at four random positions around the margin of the blastodisc, and start migrating dorsally during gastrulation. Subsequently, they move toward the intermediate targets around somites 1–3 at 10.5 hours post fertilization (hpf), and then to the final target region at somites 8–10 at 13 hpf. At 24 hpf, PGCs localize around the junction between the yolk ball and yolk extension in the gonadal region, forming compact clusters.

PGCs migrate in distinct steps in response to the chemoattractant signals provided by the cytokine SDF1A secreted by somatic cells and sensed by its receptor CXCR4B expressed on the PGCs. During these steps, other somatic cells express CXCR7B and sequester SDF1A by endocytic uptake [13], which results in the proper SDF1A gradient necessary for guiding the PGCs precisely in their migration. Although the exact molecular mechanism that signals the end of migration to the PGCs is still unclear in zebrafish, regions of high SDF1A expression seem to dictate where they terminate their migration. It has also been suggested by means of transplantation experiments that the migratory activity of PGCs seems controlled autonomously, and that the ability to migrate is not restored by changes in the environment [14]. Once the PGCs reach the gonads, they begin to form tighter clusters around 10 days post fertilization (dpf) when compared to previous stages, suggesting that PGCs themselves change their developmental phase [14].

**Figure 1:**
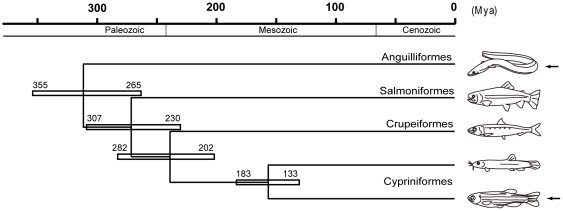
Times of divergence among bony fishes (modified from Inoue et al., 2005 [**17**] and Peng et al., 2006 [**18**]), presented simply as the average of the various estimates. The width of the horizontal rectangles reflects the extent of variation among the different estimates. The time of divergence between Anguilliformes and Cypriniformes (arrows) was estimated to be 265–355 mya (around the Paleozoic period).

On the other hand, the migration pattern of PGCs has been shown to differ among various teleost species [15]. It appears reasonable to assume that this variation in the PGC migration patterns reflects the differences in egg size, shape, yolk-composition, developmental period, etc., in a diverse group such as Teleostei that is widely dispersed among various environments and ecological niches almost all over the hydrosphere, with a wide range of reproductive strategies. Therefore, it would be interesting to investigate how PGC migration patterns and mechanisms are modified among species that have adopted a large variety of reproductive strategies and developmental patterns. Studying PGC migration at the molecular level in various fish species will also help us understand the evolution of this process among them. One simple and direct approach would be to do an interspecific PGC transplant and observe its behavior after transplantation. In cyprinid species in which the migration patterns of PGCs are relatively similar, it has been already shown that a single PGC is transplantable [14], [16]. The main goal of this investigation was to study, by means of transplantation, if the main function of migration towards the gonads is conserved across distantly related fishes: Japanese eel, *Anguilla japonica*, (family Anguillidae), and zebrafish (family Cyrinidae), which are estimated to have diverged 265–355 million years ago based on a partitioned Bayesian approach (Figure 1) [17], [18]. We isolated and transplanted individual eel PGCs into zebrafish embryos and observed their behavior, which we report in this paper.

The Japanese eel is a species found in Japan, Korea, Vietnam, the East China Sea and the northern Philippines. The reproductive behavior, spawning grounds, and embryonic development of this fish were largely unknown until recently and have attracted a great deal of interest. However, several aspects of the fish still remain unclear. For example, the mechanism of the migration of eel PGCs and gonadal development are still unknown, primarly due to the difficulty of collecting embryos and young fish before the transformation of a leptocephalus into a round glass eel. Japanese eel is not only commercially important as food fish, but it also happens to be interesting and attractive material from the view point of evolutionary biology as well. For example, it has been suggested by means of phylogenetic analysis using whole mitochondrial genomes that freshwater eels originated from the midwaters of the deep ocean [19].

## Results

### Visualization of eel PGCs by injecting GFP-*nos*1 3′UTR mRNA

**Figure 2:**
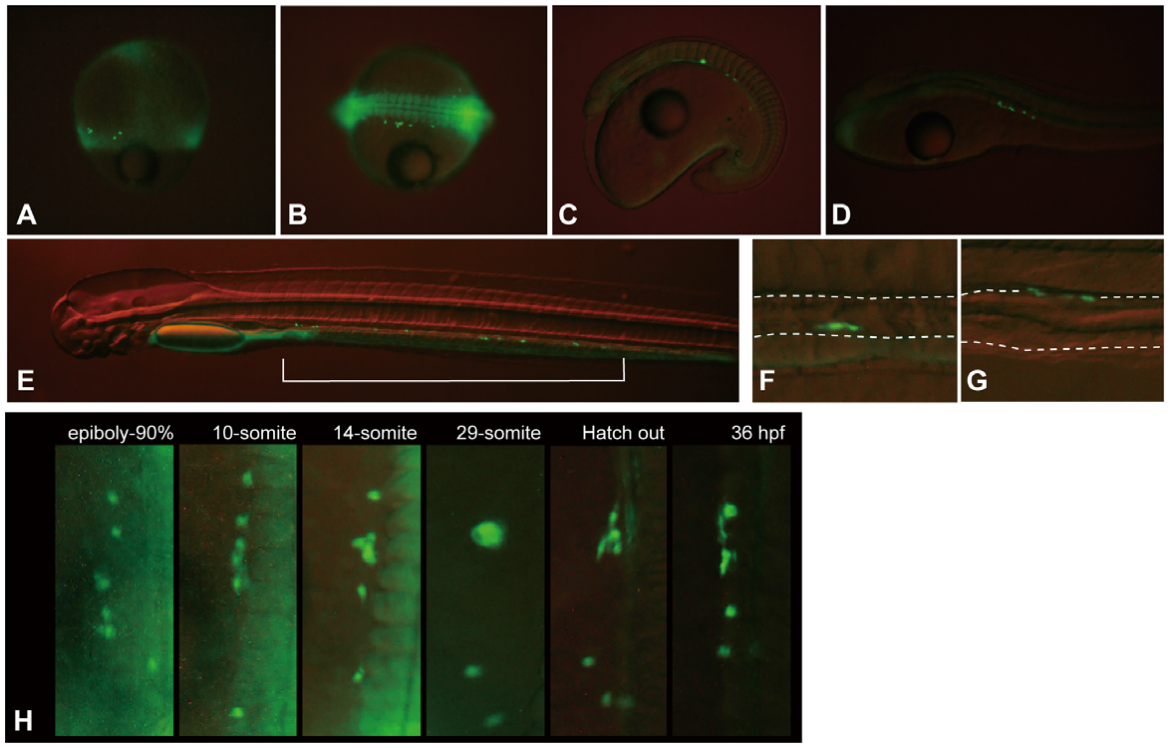
Localization and behavior of visualized eel PGCs during migration. (A) 60%-epiboly stage. (B) 14-somite stage. (C) 29-somite stage. (D) Embryo at 36 hpf. (E) Embryo at 84 hpf. (F) A magnified image of Figure 2E, where PGCs were located. PGCs were localized around the lower side of the developing gut. (G) A magnified image of 6 dpf embryo, where PGCs were localized. GFP-labeled PGCs were seen localized toward the upper side of the alimentary canal. (H) The coalescence of PGCs during their migration (also see Movie S1). These figures show that several PGCs coalesced tightly together during somitogenesis with the appearance of almost a single cell. The PGCs, however, broke apart and proceeded with migration at around the time of hatching. The bracket in E shows the area where PGCs were localized in the embryo. Dashed lines in F and G delineate the outline of a developing alimentary canal.

In this study, our first objective was to investigate the normal route of PGCs migration in eel embryos. Therefore, we injected GFP-*nos*1 3′UTR mRNA (300 ng/µl in 0.2 M KCl) into 1–2 cell stage eggs, with three replications. In total, 292 eggs were injected with the mRNA and 135 embryos developed normally at two days post fertilization (dpf), of which 80 (59.3%) contained cells that showed strong GFP expression (Table 1). The average number of GFP-positive cells in these embryos was 5.2 (SD: 2.5; Range: 1–11), and up to nine GFP-positive cells were localized in one side of the body only, in as many as 44 embryos (55%), suggesting an unequal distribution of mRNA within these embryos. As in other teleosts, these cells first appeared at the margins of the blastodisc, and migrated toward the lateral side of the developing embryonic body during somitogenesis. GFP-expression in these cells was up-regulated as the embryos developed, although the background GFP-expression was drastically reduced during development in other somatic cells (Figure 2A–E). In addition, it was clear that the 3′UTR of the *nanos*1 gene, well-known as a germ-line specific marker, could function only in GFP-positive cells. It has been already shown that the 3′UTR is subject to degradation in somatic cells, but is stabilized in PGCs by interaction with the microRNA, miR-430 [20], [21]. Therefore, we considered GFP-positive cells to be PGCs, and refer to them as such henceforth in this paper.

**Table 1:** Number of embryos with GFP-labeled PGCs at 1 day post fertilization after GFP-*nos*1 3′UTR mRNA injection.

Trial	Exp. group	No. of embryos	No. of normal embryos	No of embryos with PGCs
Exp.1	injected	144	50 (34.7)	33 (66.0)
	control	90	80 (88.9)	-
Exp.2	injected	78	49 (62.8)	35 (71.4)
	control	44	40 (90.9)	-
Exp.3	injected	70	36 (51.4)	12 (33.3)
	control	52	44 (84.6)	-
				
Total	injected	292	135 (46.2)	80 (59.3)
	control	186	164 (88.2)	-

In order to understand the migration pattern of eel PGCs in detail, 32 embryos were individually observed every two hours, photographs taken and the location of the PGCs identified. Additionally, time-lapse movies of three embryos were taken and the behavior of the PGCs recorded during their migration. The PGCs were first observed around the marginal and dorsal regions of the blastodisc at approximately the 50%-epiboly stage, although these cells were not observed at the animal pole and cleavage cavity (Figure 2A). At the 10-somite stage, almost all these cells were localized at the lateral sides of the dorsal axis from the head to the vicinity of the yolk plug, although at times a few PGCs were observed at the head, trunk, or ventral regions, where they remained for at least one week. During the 15–35 somite stage, the PGCs continued to migrate but began to localize around similar regions in the lateral sides of the trunk, although the region was gradually restricted to the side of the somites (Figure 2B–D). During this period, some PGCs aggregated and formed big cluster(s) that appeared almost as a single cell (Figure 2H and Movie S1). Later, these clusters separated with no change in the original number. The length of the period for aggregation depended on the embryo. At 5-dpf the PGCs were localized at the ventro-lateral sides of the newly formed alimentary canal (Figure 2E, F), and at 7-dpf, these cells moved toward the dorsal side of the canal (Figure 2G). At these stages, they were still distributed widely along the alimentary canal and did not aggregate at any specific position, as seen in Figure 2E (indicated by the bracket). This distribution pattern lasted at least until 10-dpf. The average number of GFP-labeled PGCs in a given embryo at hatching was 5.6. We were not able to trace the migration of these cells after 10-dpf because our lab is not equipped to maintain the embryos for extended periods of time.

### Transplantation of eel PGC into zebrafish embryos

**Figure 3:**
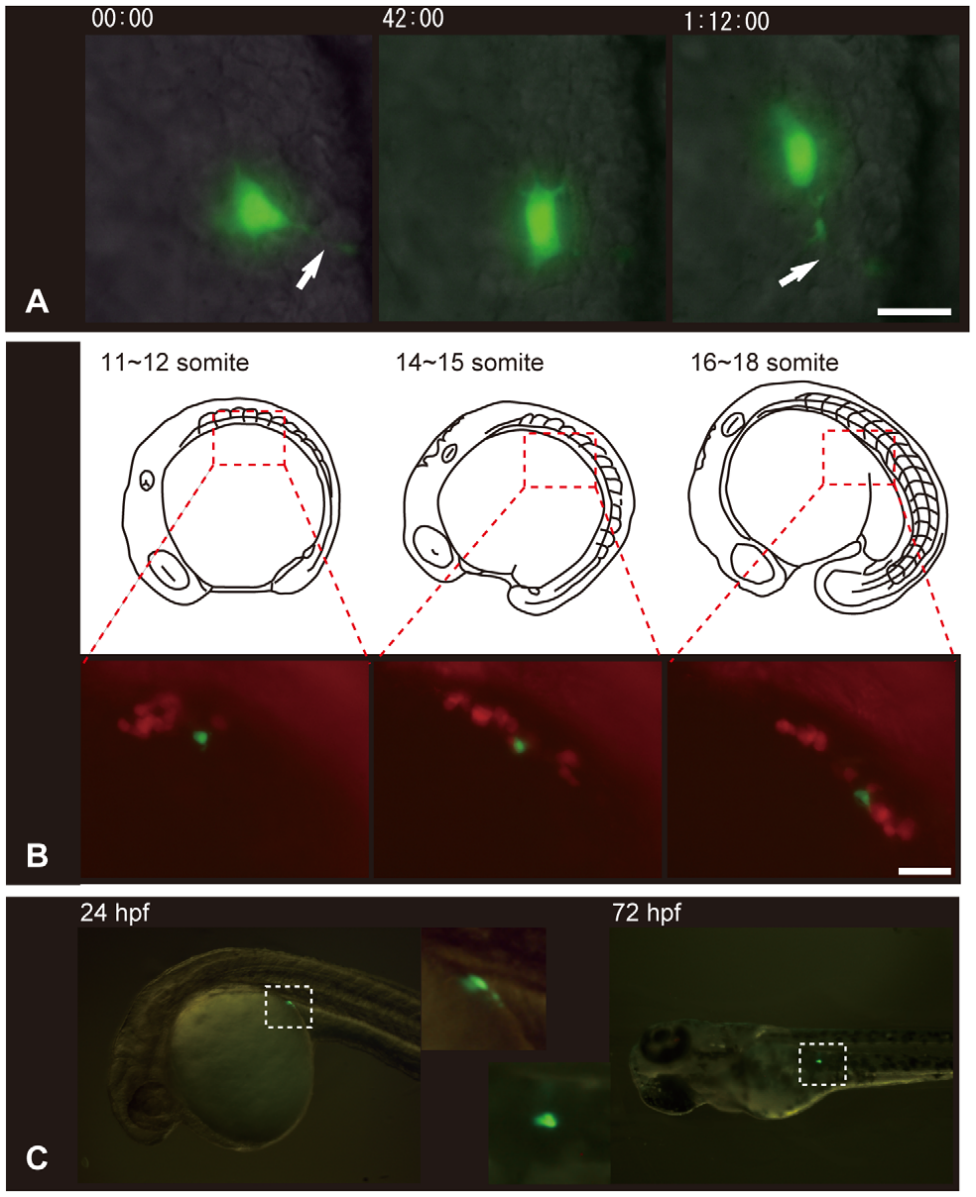
Behavior and migration of a transplanted eel PGC in zebrafish host embryo. (A) An eel PGC extended a long filopoduim-like process and moved actively in zebrafish embryos (also see Movie S2). The time elapsed (in minutes) from the time imaging was begun can be seen at the top of each figure. Arrows indicate the filopodium-like process. (B) A transplanted eel PGC migrated along with endogenous zebrafish PGCs toward the area of future gonad formation. The GFP-labeled cell is a transplanted eel PGC and RFP-labeled cells are endogenous zebrafish PGCs (also see Movie S3). Boxed areas with red dashed lines in the upper illustration indicate the region in the corresponding photograph. (C) A transplanted eel PGC that has migrated to the precise region of future gonad formation in the zebrafish embryo. The two smaller images in the middle show the corresponding boxed areas at a higher magnification. The scale bars in A and B represent 10 µm and 50 µm, respectively.

Eel PGCs could be visualized with GFP under the fluorescent stereo-microscope**,** as described above. It has already been shown that it is possible to transplant a single visualized PGC into other embryos and produce germ-line chimera [14], [16]. Using this technique, we transplanted a GFP-labeled eel PGC into zebrafish blastula embryos, and time-lapse images of the PGC were taken every 2 minutes during the development. After transplantation, the eel PGC extended its pseudopodia and moved actively around the region where the zebrafish PGCs gather: the lateral sides of the somites (Figure 3A and Movie S2). To investigate whether the eel PGC correctly recognized the guidance signals for zebrafish PGCs during migration, the host fish PGCs were labeled with RFP by injecting RFP-*nos*1 3′UTR mRNA, and GFP-labeled eel PGC was then transplanted into the host. In the chimeric embryo, at around the 10-somite stage, the eel PGC clustered with the zebrafish PGCs, and joined them in their migration toward the gonadal region (Figure 3 and Movie S3 **)** in 42.7% of the empryos (Table 2). The eel PGC was seen in the gonadal region for 6–7 days and disappeared in many embryos around the time when the eggs hatched and the hatchlings began to feed. A few fish retained the PGC in the gonadal region even after this period of initial feeding, but it did not proliferate (n = 6).

**Table 2:** Migration efficiency of eel PGCs in zebrafish embryos.

Experiments	Total number of chimeras	No. of normal embryos	No. of embryos with PGC at day 2	Location of PGC in embryo on Day-2	
				Gonadal region (%)	Ectopic
*E-to-Z SPTC* *(Sum of 4 exp.)*	81	75 (92.6%)	51 (68.0%)	32 (42.7%)	19 (25.3%)
*Host control*	124	115 (92.7)	-	-	-

## Discussion

### Migration of eel PGCs

In this study, we visualized eel PGCs by injecting GFP-*nos*1 3′UTR mRNA. Compared to the other fishes [15], the efficiency of PGCs visualization in eel embryos was not high (59.3%), and it varied among experiments. In some embryos, PGCs were localized at only one side of the body, and only up to nine cells were visualized in each embryo. This result suggests that not all PGCs were visualized in these embryos by means of injecting the synthesized mRNA. This may be due to difficulties associated with injecting mRNA into eel embryos rather than any difference in gene function among species. Eel eggs orient animal pole to the bottom in the sea water and ringer's solution, because of the free-floating nature of the eggs as a result of the oil droplets localized in the yolk cells. This makes it difficult to inject mRNA correctly into the blastodisc where PGCs are formed, and the embryos in which the PGCs were not visualized may well have been victims of this difficulty. In this connection, it is important to note that GFP was expressed only in the yolk ball in some embryos in which PGCs were not visualized (data not shown). However, eel PGCs were successfully visualized in about 60% of the embryos. This result clearly shows that the mechanism of *nanos*1 3′UTR, subject to degradation in somatic cells and stabilization in PGCs by interaction with the microRNA, miR-430 [20], [21], is conserved between these two distantly related fish species, as previously shown among other species [15].

GFP-labeled eel PGCs appeared after the 50%-epiboly stage and these cells located to the lateral sides of the somites along the anterior-posterior axis at around the somitogenesis period. The PGCs generally appeared to stay apart from each other during migration. However, time-lapse photography revealed that some of them came together and coalesced to form a compact cellular mass that broke apart into individual cells again. In mouse, zebrafish and *Drosophila*, it has already been suggested that PGC-PGC adhesion mediated by the regulation of the adhesion molecules E-cadherin has an important role in initiating PGC migration [22]. Downregulation of E-cadherin levels in PGCs leads to their dispersal and the initiation of migration [23], [24]. Furthermore, it seems likely that motile behavior of PGCs is suppressed by cell-cell contacts between PGCs and somatic cells in *Drosophila*, because DE-cadherin and Fear of intimacy (FOI), a zinc transporter, are required for gonad coalescence and compaction of PGCs in this species [25], [26]. These comparisons suggest that cell adhesion has an important role for both initiating and terminating PGC migration. In eel embryos, however, tight adhesion occurs during migration and these cells proceed with their migration after the cells form a coherent mass. Although the mechanism and role of the adhesion in the eel embryo are still unknown, the behavior of these cells implies PGC-PGC interaction during migration.

We also found that eel PGCs did not aggregate at specific regions of the embryo. In zebrafish, PGCs migrate toward the position on the junction of the yolk ball and yolk extension where PGCs locate side-by-side, and these cells associate with gonadal somatic cells, which later form the gonads [14]. As in the zebrafish embryo, PGCs aggregate in a specific area in other model species too**,** such as *Drosophila*, chicken and mouse. In this connection, germ cells were observed on the dorsal wall of body cavity in a line widely separated from the front to the back in the gonadal rudiment, in the glass eel. Therefore, it is likely that this localization pattern of primordial germ cells in the embryo is retained till a later stage, suggesting that gonadal development in eel is different from that of other model species, such as zebrafish.

**Figure 4:**
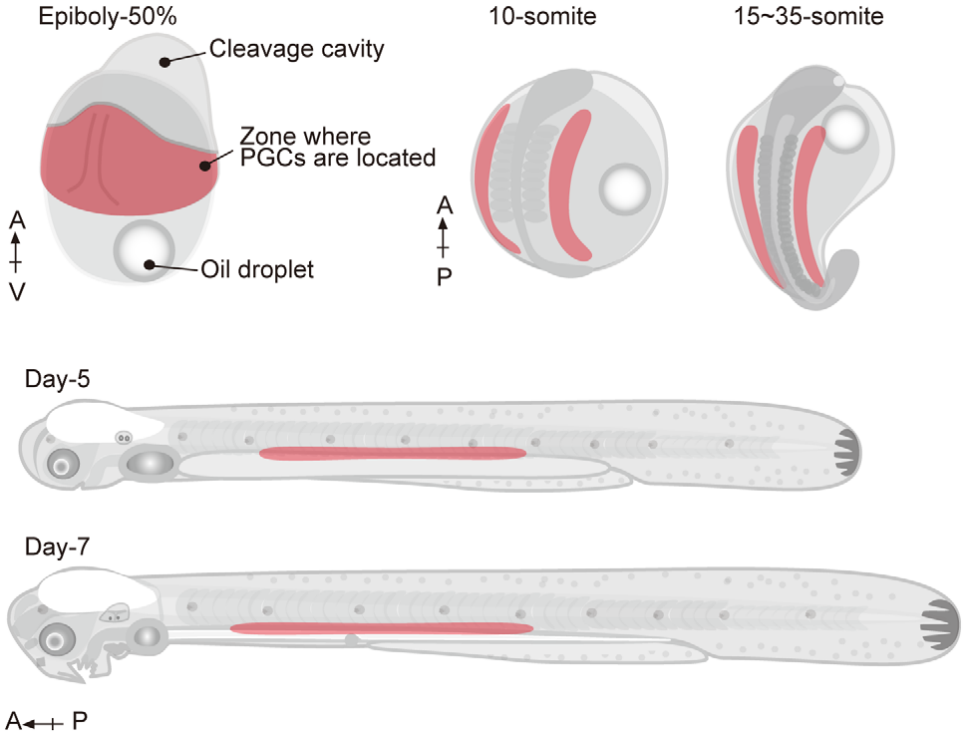
A schematic illustration of the localization of eel PGCs during embryonic development. Localization of PGCs in embryos at each stage is shown by means of dots, and is summarized in this illustration. The regions where PGCs were well observed are colored red. At the 50%-epiboly stage, PGCs were found widely around the embryonic shield. During the somitogenesis period, they were localized bilaterally and spread loosely by the somites. During this period, some of the PGCs were observed to first coalesce and then disperse once more. The PGCs finally localized around the upper part of the gut; these cells did not form clusters.

We have summarized the distinctive characteristics and localization patterns of eel PGCs in the embryos that emerged from our data, and compared these characteristics to the PGC migration in zebrafish (Figure 4 and Table 3). It can be seen that the localization patterns, cell shape, and behaviors of each PGC are very different between Japanese eel and zebrafish. When an eel PGC was transplanted into zebrafish embryos, however, it efficiently migrated toward the gonadal region of the host embryo, by spreading filopodium-like processes, with intermediate targets supplied by somatic cells of the zebrafish embryo with its own PGCs. This result gives evidence that the migration mechanisms of PGC are highly conserved between these two fishes irrespective of superficial differences between them. For example, the guidance mechanisms of PGCs supplied by somatic cells are conserved between the two species, strongly suggesting that chemoattractant signals by means of CXCR4B and SDF1A are also employed in the Japanese eel embryo. On the other hand, almost all the transplanted PGCs disappeared in the host fry after hatching. Transplanted loach/goldfish PGCs differentiate into functional sperm in zebrafish hosts, suggesting that the molecular mechanisms of PGC migration and differentiation to sperm are conserved among cyprinids [16]. On the other hand, functional eggs are not differentiated in these germ-line chimeras, suggesting that the mechanisms of egg differentiation are different between loach and zebrafish. Taken together, these facts suggest that the development of germ cells in eel and zebrafish is different in the early stages compared to the cyprinids, and that these differences make it difficult for the transplanted cells to develop into more advanced developmental stages after 6–7 days. However, since almost all the control eel embryos were also dead in our study until 10 dpf, it is not clear whether this result comes from eel cell's own mortality or an incompatible donor/host combination of germline chimera.

**Table 3:** Behavioral differences between eel and zebrafish PGCs during migration in the host embryo.

	Japanese eel	Zebrafish
Behaviors of PGCs during migration	• Cells generate filopodium -like process.• Some cells coalesce tightly during migration.	• Cells generate membrane blebbing.• PGCs move as clusters toward the region of future gonad formation, but do not coalesce (unlike in eel).
Final position of PGCs in embryo	• Localized separately and widely	• Around the junction between yolk-ball and yolk-extension

Surrogate production, where germ-line chimera are produced by transplanting germ-cells, has been attracting attention lately, because this technique can help produce gametes of endangered species using more common species as the surrogate host [27], [28]. The key step in producing xenogeneic germline chimeras is to make donor germ cells incorporate into the host gonad. In this study, we have demonstrated that PGCs isolated from eel embryo were able to migrate toward the gonadal region of zebrafish. This result indicates that germline chimera can be produced between distantly related species – even when one of which spawns in the sea and the other in freshwater. This data suggests that it may be possible to produce marine fish gametes using freshwater species using appropriate species combinations of donor and host, as previously suggested by Yamaha et al (2007) [28]. Generally, however, it is more difficult to keep marine fish in artificial containers when compared to freshwater fish because the salinity of sea water is easily changed by the effect of condensation and evaporation at the surface. In freshwater fishes, production of target gametes by means of germ cells transplantation between different species has been more feasible [16], [29]–[31]. Therefore, it might be worthwhile to transplant PGCs to obtain marine fish gametes through freshwater hosts, as shown in this study.

## Materials and Methods

### Ethics

All experimental procedures were performed in accordance with National and Institutional guidelines on animal experimentation and care, and were approved by the Animal Research Committee of Hokkaido University (approval ID: 22-1).

### Preparation of embryos

Parent zebrafish were maintained at 26 to 28°C under a 16 hour light/8 hour dark photoperiod at the Nanae Fresh Water Laboratory, Hokkaido University. Fertilized eggs were obtained during the light period by means of natural mating: one female and two males were placed together in a 10 liter fish tank at 26∼28°C. Embryos were dechorionated with 0.1% trypsin (Difco) and 0.002% actinase E (Kaken) in Ringer's culture solution (128 mM NaCl, 2.8 mM KCl, 1.8 mM CaCl_2_), and cultured in Ringer's culture solution containing 0.01% penicillin and 0.01% streptomycin. They were initially cultured at 28.5°C in 96-well plates (Greiner) individually filled with Ringer's culture solution for 24 hours, and subsequently in 24-well plates (Greiner) filled with the culture solution (1.8 mM CaCl_2_, 1.8 mM MgCl_2_) containing the same antibiotics as above. The stages of embryonic development were identified according to Kimmel et al., 1995 [32].

Japanese eel were kept in Nansei station, National Research Institute of Aquaculture, Fisheries Research Agency and Faculty of Fisheries Sciences, Hokkaido University. Maturation in both sexes was induced by injecting salmon pituitary extract. Fertilized eggs were obtained by means of artificial insemination, following Ohta et al., 1997 [33].

### Construction and synthesis of mRNA

Capped sense GFP-*nos*1 3'UTR RNA was synthesized in vitro using the mMESSAGE mMACHINE kit (Ambion). The mRNA was prepared into 300 ng/µl with 0.2 M KCl before injecting.

### Microinjection of mRNA

For the purposes of observation and transplantation of PGCs, GFP-*nos*1 3UTR mRNA was injected into Japanese eel eggs around the blastodisc region during the 1-4 cell stage without removing the chorion in the Ringer's culture solution. Ringer's solution was used to ensure that the floating embryos sank to the bottom of the dish. Injected embryos were cultured in sterilized sea water containing antibiotics until observation or preparation for collecting PGCs.

### Observation of Japanese eel PGCs

Just before the observations were made, chorions were manually removed, using a pair of fine forceps, from embryos in Ringer's solution containing antibiotics, and the naked embryos were placed on an agar coated glass dish and suitably aligned for taking photographs. The embryos were observed and photographed using a fluorescent stereo-microscope (Leica MZ16F) equipped with a digital camera (Leica DFC300FX). The images captured under two different fluorescence spectrums, GFP and RFP, were merged into one image using Adobe Photoshop CS3 software.

### Time-lapse imaging

mRNA injected eel embryos with GFP-labeled PGCs were placed in a 60 mm dish filled with 3% methylcellulose (Sigma) in sterilized 50% sea water for time-lapse imaging (Leica inverted microscope and softwere LAS). Chimeras generated by a single PGC transplantation were placed in a 60 mm dish filled with 3% methylcellulose in Ringer's solution for the same imaging procedures. Images were taken every 2 minutes at room temperature, and these serial images were then converted into an animation using Leica imaging software (LAS).

### Transplantations

Japanese eel PGCs were transplanted during early somitogenesis into the blastula of zebrafish, following Saito et al., 2008 [16]. The procedure is briefly summarized here. GFP-positive PGCs were dissociated from labeled embryos during somitogenesis using 1% citric acid trisodium and 0.1% collagenase (Wako) in Ringer's solution and gentle pipetting. The dissociated cells were transferred into 120 mm glass dishes filled with 5% FBS (Gemini bio-products) in Ringer's solution containing 0.01% penicillin and 0.01% streptomycin. Isolated PGCs were identified as GFP-positive cells and aspirated into a glass microneedle under a fluorescent stereo-microscope. A single PGC was transplanted into the marginal region of the blastodisc of each blastula stage zebrafish embryo. The migration efficiency was determined as the ratio of the number of embryos with PGCs located at the gonadal region to the number of embryos that developed normally at 24 hpf.

## Supporting Information

Movie S1 **The coalescence of PGCs during their migration.** In this movie, two PGCs coalesced tightly together during somitogenesis with the appearance of almost a single cell.
(MOV)

Movie S2 **An eel PGC extended a long filopoduim-like process and moved actively in zebrafish embryo.**
(MOV)

Movie S3 **A transplanted eel PGC migrated along with endogenous zebrafish PGCs toward the area of future gonad formation.** The GFP-labeled cell is a transplanted eel PGC and RFP-labeled cells are endogenous zebrafish PGCs.
(MOV)
